# Percutaneous pinning versus pin-in-plaster for treatment of distal radius fractures

**DOI:** 10.22088/cjim.10.3.309

**Published:** 2019

**Authors:** Farhang Tajeddin, Seyyed Mokhtar Esmaeilnejadganji, Behnam BaghianiMoghaddam, Sekineh Kamali Ahangar, Masood Bahrami Feridoni

**Affiliations:** 1 Student Research Committee, Babol University of Medical Sciences, Babol, Iran; 2 Mobility Impairment Research Center, Health Research Institute, Babol University of Medical Sciences, Babol, Iran; 3 Cancer Research Center, Health Research Institute, Babol University of Medical Sciences, Babol Iran; 4 Department of Orthopedic Surgery, School of Medicine, Babol University of Medical Sciences, Babol, Iran; 5 Clinical Research Development Center, Shahid Beheshti Hospital, Babol University of Medical Sciences, Babol, Iran

**Keywords:** Distal radius fracture, Percutaneous pinning, Pin-in-plaster, Fracture

## Abstract

**Background:**

Distal radius fractures constitute 17% of all fractures and 75% of forearm fractures in adults. Due to the vital role of the hands in a wide range of daily tasks, quick recovery and fewer complications for the patients are important. The purpose of this study was to compare the two common treatments of distal radius fractures namely, percutaneous pinning and pin-in-plaster.

**Methods:**

An observational analytical cohort study was conducted on 74 patients with distal radius fracture treated with percutaneous pinning fixation and pin-in-plaster techniques. The patients, aged more than or equal to 18 years with unilateral, closed and unstable distal radius fractures were treated in Shahid Beheshti Hospital during 2007 to 2010.The data were entered into the SPSS Version 20 statistical software and analyzed by student’s t-test, one-way ANOVA and repeated measures test.

**Results:**

Randomly, 31 patients were in pin-in-plaster group and 43 patients in percutaneous pinning group. The patients’ age range was 18-74 years. The average of radial inclination and palmar tilt after 6 weeks was significant (P=0.02, p<0.0001) in patients with percutaneous pinning. The performance of the patients after 3 and 12 months in both groups was significant (p=<0.0001).

**Conclusion:**

This study revealed that in approach to unstable DRF in adults, PCP method shows better improvement compared to PP technique.

Distal radius fracture (DRF) constitutes 17% of orthopedic fractures and 75% of forearm fractures in adults (1-3). Most of these fractures occur in elderly persons and postmenopausal women (4, 5). The leading causes of fractures in these individuals are high-energy trauma and osteoporosis (6, 7). The objectives of treatment are appropriate anatomical reduction, bone stabilization during fracture healing process and regaining wrist movements (8). Complications of DRF include restriction of wrist movement and forearm rotation, complex regional pain syndrome (CRPS) and nerve damage (1, 9). Selecting the proper treatment method is still an important issue among orthopedic surgeons (10). Various methods containing percutaneous fixation (closed reduction percutaneous pinning), external fixation (pin in plaster) and the different types of internal fixation are used (11). Percutaneous pinning is one of the earliest techniques for DRF fixation. This low-cost and minimally invasive technique can be used in extra-articular fractures and intra-articular without significant communication. Its advantages consist of corrigibility, lightness, easy access to the wound site and better preservation of radial length (12-15).

Pin in plaster technique can be performed in different ways (16). With advancement of these methods, better limb function and short time recovery can be provided for the patients (12). Fracture union is not the only purpose of DRF treatment, but appropriate performance, radiographic and clinical outcomes, and also patient satisfaction are considered (17). Therefore, an ideal treatment includes correction and maintenance of radial length, radial inclination and palmar tilt (14). Since the upper limb is an essential organ and is involved in the most movements and actions, rapid improvement and fewer complications in its fractures play a vital role for the patients. The aim of this study was to compare percutaneous pinning and pin in plaster as a treatment of DRF.

## Methods

An observational analytical cohort study was conducted on 74 patients with distal radius fracture who were treated with percutaneous pinning (PCP) and pin-in-plaster (PP) techniques. The patients with unilateral, closed and unstable intraarticular distal radius fractures received treatment in Shahid Beheshti Hospital during March 2007 to February 2010. In this study, oblique volar fractures, open fracture, bilateral fractures, and multiple fractures were excluded. Also, because of the different pediatric orthopedic approach, the patients aged less than 18 years were excluded. Randomly, thirty-one patients were in PP group and 43 patients in PCP group. Informed consent was obtained from all the patients. Patient information includes age, gender, smoking status, side of fracture, fracture mechanism, type of fracture, radiographic survey consisting radial length, radial inclination and palmar tilt and upper limb functions (wrist supination, pronation, flexion, extension, radial deviation and ulnar deviation) (6 weeks, 12 weeks and 12 months post-operation in anterior-posterior (AP) and lateral views). Fractures were classified based on Frykman classification. 

Both groups of patients were operated in supine position, with general anesthesia and after 10-minute scrub. In PP group, one pin was placed in the base of metacarpal bone and another in the styloid process of radius. After reduction and traction, casting was done. Then the radiographies of both wrists were performed to compare intact and injured limb. Further radiographies of both hands were performed after 6 weeks; then plaster and pins were removed and short arm cast was applied for 4 weeks. In PCP group, following anesthesia and scrub, a 20^_^centimeter radius cotton roll was placed under the wrist and reduction was performed by traction and manipulation. Two parallel pins were inserted through styloid process to proximal part of radius, while the wrist was in plantar flexion. Pins with 1.5 to 2 millimeter diameters were used. 

Long arm cast was applied after 48 to 72 hours of long arm splint application. Six weeks postoperatively, radiographs were obtained from both wrists and plaster was removed. An expert evaluated all X-ray images by means of radiological criteria and orthopedic ruler. Both groups had been followed-up for 12 weeks and 12 months after the surgery and hand function was assessed in terms of supination, pronation, flexion, extension, radial deviation and ulnar deviation. 

Data analyses were performed using SPSS Version 20 statistical software (SPSS Inc. Chicago, IL, USA) using Student’s t-test, one-way ANOVA and repeated measures test, p<0.05 was considered significant.

## Results

Seventy four patients were enrolled in the study, 31 in PP group and 43 in PCP group. The age range of patients was 18 to 74 years. Patient information includes age, gender, smoking status, injury mechanism and fracture side which are given in Table-1.

**Table 1:** Baseline characteristics in both groups

**Variable**	**PP**	**PCP**	**P value**
Age(M±SD) Male Smoking	36.84±13.76 20(64.5) 12(38.7)	41.70±14.63 18(41.9) 12(27.9)	0.15 0.04 0.45
Side(Right)	10(32.3)	17(39.5)	0.62
Mechanism of injury			
Accident Falling Falling from height Others	14(45.1) 9(29.0) 6(19.3) 2(6.4)	9(20.9) 25(58.1) 8(18.6) 1(2.3)	0.001

Fractures were classified according to the Frykman classification. In PP group, type VIII and in PCP group type II constituted the largest number, but there was no statistically significant difference (Table-2).

**Table 2:** Fracture classification according to Frykman classification

	**PP (%)**	**PCP (%)**	**P value**
Frykman classification			0.09
I	5(16.2)	4(9.3)	
II	3(9.7)	15(34.9)	
III	3(9.7)	4(9.3)	
IV	1(3.2)	-	
V	4(12.9)	4(9.3)	
VI	3(9.7)	3(6.9)	
VII	2(6.4)	7(16.3)	
VIII	10(32.3)	6(14.0)	

Mean radial inclination and palmar tilt relative to normal condition at 6 weeks, 12 weeks and 12 months was significantly different in PCP patients compared to PP patients (Table-3).

**Table 3:** Average of variables after reduction

**Variable**	**PP** **M±SD**	**PCP** **M±SD**	**P value**
**Radial length(mm)**			
Healthy Hands	14.45±3.78	14.40±4.24	0.95
6 weeks	12.13±3.67	13.30±4.34	0.22
12 weeks	12.29±3.85	13.21±4.35	0.35
12 months	13.09±4.23	12.16±3.90	0.33
**Radial Inclination(Grade)**			
Healthy Hands	22.97±3.39	22.37±3.94	0.96
6 weeks	19.61±4.55	21.72±3.52	0.02
12 weeks	19.03±4.59	21.12±3.73	0.03
12 months	18.81±4.46	20.91±3.79	0.03
**Palmar tilt(Grade)**			
Healthy Hands	7.32±4.36	6.60±4.75	0.5
6 weeks	-2.55±4.91	3.16±6.04	<0.0001
12 weeks	-2.58±4.95	2.37±7.04	<0.0001
12 months	-2.68±4.95	2.70±53.92	<0.0001

The function of both hands was evaluated in both groups at 3 and 12 months. Supination, pronation, flexion, extension, radial deviation and ulnar deviation were significantly different in two groups (P<0.0001) (Fig 1). In the first group, one case was operated twice because of osteomyelitis in the upper pin location. Infection in another group occurred as superficial cellulitis in two cases that was cured with antibiotic therapy.

## Discussion

The patients who were treated with PCP method had better radiologic and functional improvement rather than those treated with PP method. Radiologic improvement included radial inclination and palmar tilt. Radial length has no statistical significant difference between groups. Functional improvements include patients’ ability to attain supination, pronation, radical deviation, ulnar deviation, wrist flexion, and wrist extension movement angles.

 Radiologic improvement is an important aim in the treatment of DRF. Clinical judgment for treatment process depends on radiology. Our results showed that patients benefited from PCP method more than the PP method. There are some studies that indicated PCP method leads to better improvement similar to our results. Brian et al. have used percutaneous pinning and external fixation in a study performed in 2004 on 50 patients with DRF. The range of patients’ age was 19 to 62 years. The distribution of fractures in two groups was similar. Radial length, radial inclination and palmar tilt were significantly different in percutaneous pinning group in comparison with external fixation group (18). In a study entitled “Outcomes of Pin and Plaster Versus Locking Plate in Distal Radius Intra-articular Fractures” in 2013, Bahari-Kashani et al. found that radial length, radial inclination and palmar tilt showed better improvement in locking plate group (1). A study of Mansouri et al. in 2006 on 132 DRF patients aged 17 to 72 years demonstrated that percutaneous pinning radiological criteria including radial length radial inclination and palmar tilt had significant improvements (8). Chin-En Chen et al. in 2008 found that PCP technique is more effective for the improvement of DRF rather than PP technique. They concluded this finding from the radiologic assessment of patients with DRF including radial length and volar tilt(19).

The patients with DRF are so concerned about their ability to move the wrist. So another important aim of DRF treatment is the functional ability of patients to do different movements of the wrist including supination, pronation, radial deviation, ulnar deviation, flexion and extension. Each method that presented less disability for patients is preferred. In our study, PCP method leads to the patient’s less movement disability in comparison to PP. These results are similar to other studies’ findings. In 1997, Rodriguez-Merchan et al. conducted a study on 20 patients with DRF. They found that the upper limb functional criteria had significant differences between PP group and PCP group (20). Mansouri et al. in 2006 also concluded that there were significant differences in functional criteria such as supination, pronation, flexion, extension, radial deviation and ulnar deviation in patients treated with PCP technique in comparison to PP technique (8).

In conclusion, this study revealed that in approach to unstable DRF in adults, PCP method shows better compared to PP technique. Therefore, this method is recommended since it is simple and convenient. We can also prevent its complications by means of intra-operative care and experience enhancement. We also recommend designing clinical trials to evaluate the efficacy of PCP technique more specifically precise with high level evident studies. 

## References

[1] Bahari-Kashani M, Taraz-Jamshidy M, Rahimi H (2013). Outcomes of pin and plaster versus locking plate in distal radius intraarticular fractures. Trauma Mon.

[2] Alffram P, Bauer G (1962). Epidemiology of fractures of the forearm a biomechanical investigation of bone strength. J Bone Joint Surg Am.

[3] Bucholz R, Heckman J, Court-Brown C, Ruch D, McQueen M, eds (2010). Distal radius and ulna fractures, Rockwood and Green's fractures in adults.

[4] Riggs B, Melton L (1983). Evidence for two distinct syndromes of involutional osteoporosis. Am J Med.

[5] Lindau T, Aspenberg P, Arner M, Redlundh-Johnell I, Hagberg L (1999). Fractures of the distal forearm in young adults An epidemiologic description of 341 patients. Acta Orthop Scand.

[6] Cuenca J, Martínez A, Herrera A, Domingo J (2003). The incidence of distal forearm fractures in Zaragoza (Spain). Chir Main.

[7] Miller SW, Evans JG (1985). Fractures of the distal forearm in Newcastle: an epidemiological survey. Age Ageing.

[8] Mansouri M, Siavoshi B (2006). Comparison of close reduction and percutaneous pin fixation as treatments for distal metaphyseal radius fractures. Yafteh.

[9] Saeed A, Ja'afari D, Taheri H, Shariatzadeh H (2007). The effect of ulnar styloid fracture with extra-articular fracture of distal radius on rotation of wrist. Iran Univ Med Sci.

[10] Pooramiri A, Saied A, Ilka S, Ranjbar L (2013). Short-term results of using injectable bone cement technique in the treatment of unstable distal radius fractures. J Kerman Univ Med Sci.

[11] Fernandez D, Jupiter J (1996). Fractures of the distal radius: a practical approach to management.

[12] Jazayeri S, Kazemian G, Abdollahzadeh Lahiji F, Hesarikia H (2007). Cast Immobilization after pinning in distal radius fractures. Shahid Beheshti Univ Med Sci.

[13] Szabo R (1992). Comminuted distal radius fractures. Orthop Clin North Am.

[14] Green D (1975). Pins and plaster treatment of comminuted fractures of the distal end of the radius. J Bone Joint Surg Am.

[15] Dzaja I, MacDermid J, Roth J, Grewal R (2013). Functional outcomes and cost estimation for extra-articular and simple intra-articular distal radius fractures treated with open reduction and internal ixation versus closed reduction and percutaneous Kirschner wire ixation. Can J Surg.

[16] Baghdadi T, Mortazavi S, Ajvadi A (2004). Pin and plaster for distal radius fractures (a comparative study of tow techniques). Tehran Univ Med Sci.

[17] Kambouroglou GK, Axelrod TS (1998). Complications of the AO/ASIF titanium distal radius plate system (pi plate) in internal fixation of the distal radius: a brief report. J Hand Surg Am.

[18] Harley BJ, Scharfenberger A, Beaupre LA, Jomha N, Weber DW (2004). Augmented external fixation versus percutaneous pinning and casting for unstable fractures of the distal radius--a prospective randomized trial. J Hand Surg.

[19] Chen CE, Juhn RJ, Ko JY (2008). Treatment of distal radius fractures with percutaneous pinning and pin-in-plaster. Hand (N Y).

[20] Rodriguez-Merchan EC (1997). Plaster cast versus percutaneous pin fixation for comminuted fractures of the distal radius in patients between 46 and 65 years of age. J Orthop Trauma.

